# Supplementary material for: Microglia Depletion Attenuates the Pro-Resolving Activity of the Formyl Peptide Receptor 2 Agonist AMS21 Related to Inhibition of Inflammasome NLRP3 Signalling Pathway: A Study of Organotypic Hippocampal Cultures
Source: Cells. 2023 Nov 3;12(21):2570. doi: 10.3390/cells12212570 (PMC10648897; doi:10.3390/cells12212570)
Supplement: Supplementary file 1 [file cells-12-02570-s001.zip › cells-2649084-supplementary.pdf]

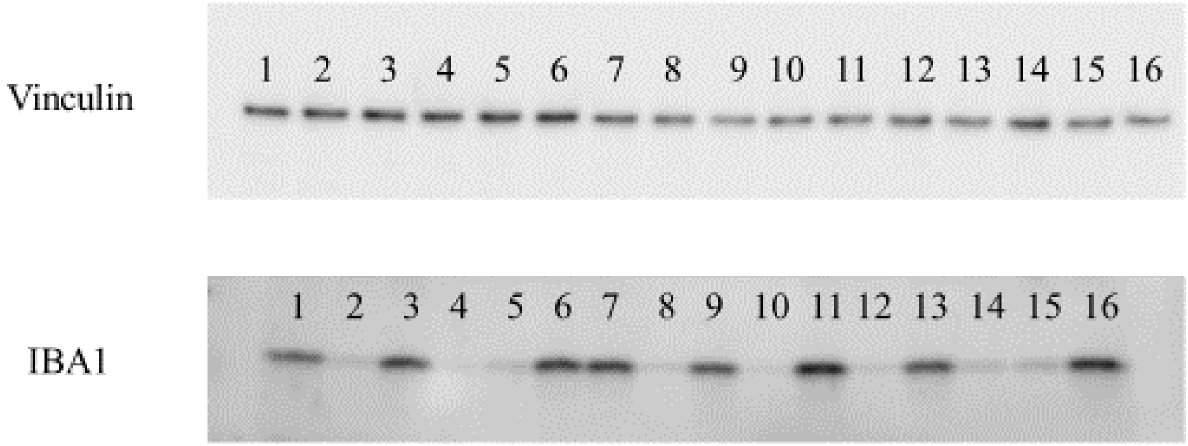

LPS 1 μg/ml	-	+	-	+
Clodronate 150 μg/ml	-	-	+	+
Sample ID	1, 3, 6, 7	9, 11, 13, 16	2, 4, 5, 8	10, 12, 14, 15
N	4	4	4	4

Supplementary Figure S1 Western blot analysis of lysates from organotypic hippocampal cultures (OHCs) demonstrating protein level of IBA1 protein. Vinculin was used as a normalizing control. Description of the arrangement of the samples (1-16).

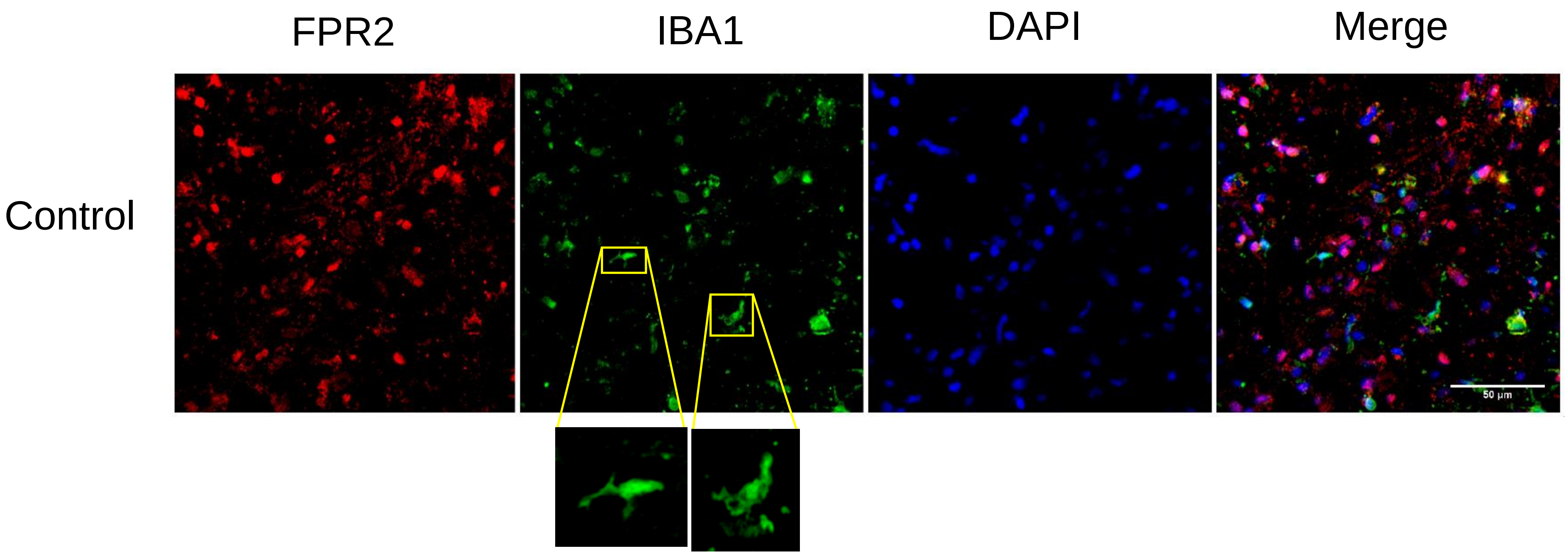
FPR2
IBA1
DAPI
Merge
Control
50 µm

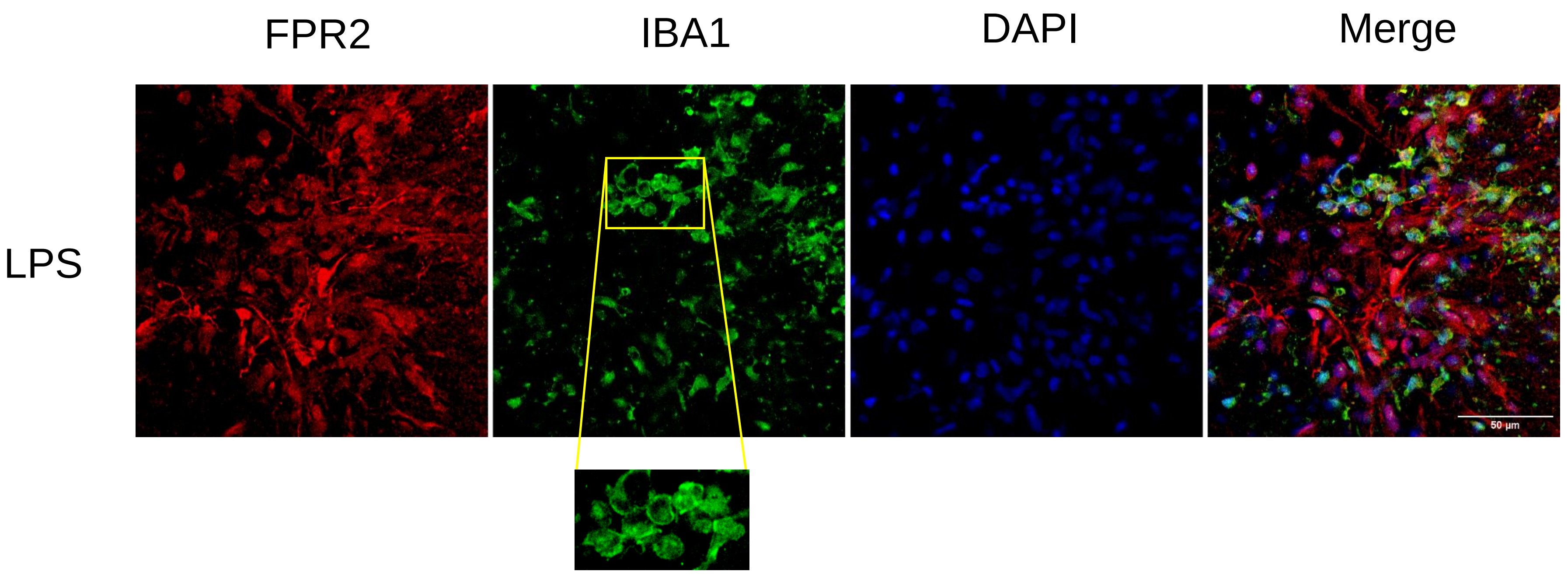
FPR2
IBA1
DAPI
Merge
LPS
50 µm
